# *Tejuino*, a Traditional Fermented Beverage: Composition, Safety Quality, and Microbial Identification

**DOI:** 10.3390/foods10102446

**Published:** 2021-10-14

**Authors:** Ángel Eduardo Rubio-Castillo, José I. Méndez-Romero, Ricardo Reyes-Díaz, Lourdes Santiago-López, Belinda Vallejo-Cordoba, Adrián Hernández-Mendoza, Sonia G. Sáyago-Ayerdi, Aarón F. González-Córdova

**Affiliations:** 1 Laboratorio Integral de Investigación en Alimentos, Tecnológico Nacional de Mexico, Instituto Tecnológico de Tepic, Av. Tecnológico No. 2595, Col. Lagos del Country, Tepic CP 63175, Nayarit, Mexico; angel.rub@icloud.com; 2 Laboratorio de Calidad, Autenticidad y Trazabilidad de los Alimentos, Centro de Investigación en Alimentación y Desarrollo A.C. (CIAD), Carretera Gustavo Enrique Astiazarán Rosas, No. 46, Col. La Victoria, Hermosillo CP 83304, Sonora, Mexico; isd.mendezromero@gmail.com (J.I.M.-R.); ricardo.reyes@ciad.mx (R.R.-D.); lsantiagolopez@outlook.es (L.S.-L.); vallejo@ciad.mx (B.V.-C.); ahernandez@ciad.mx (A.H.-M.)

**Keywords:** artisanal *Tejuino* beverage, chemical composition, volatile compounds, microbiological quality, lactic acid bacteria

## Abstract

This study aims to analyze the chemical and microbial composition and characterize volatile compounds from the artisanal and commercial *Tejuino* beverage. For this, eight samples are analyzed (four artisanal and four commercial). The chemical and microbiological quality is determined by standard methods, and volatile compounds are determined by solid-phase microextraction. Overall, the physicochemical composition and microbiological quality are higher for artisanal *Tejuino* (*p* < 0.05). The pH values were 3.20 and 3.62, and 0.76 and 0.46 meq of lactic acid for artisanal and commercial *Tejuino*, respectively. With volatile compounds analyzed, esters, benzenes, and aldehydes were predominant; meanwhile, ethanol was a volatile compound with the highest concentration for all samples. *Saccharomyces cerevisiae* and *Limosilactobacillus fermentum* were identified in artisanal *Tejuino*; yeasts of the *Pichia* genera and *Lactiplantibacillus plantarum*, for commercial *Tejuino*, and *Enterococcus* genus were identified in both samples. The characterization of both types of *Tejuino* allows us to update the information available on this important Mexican beverage. In addition, the isolation of lactic acid bacteria, as representative bacteria of both drinks, offers an area of opportunity to know the potential functionality of these bacteria in traditional fermented products.

## 1. Introduction

Corn is characterized by its high content of carbohydrates, protein, dietary fiber, B vitamins, and minerals, so it is considered that its consumption has beneficial effects on health [1]. This product, when is fermented by microorganisms and the presence of some endogenous enzymes (e.g., amylases, proteases, and phytases) reducing their carbohydrate content and improves the bioavailability of B vitamins [2]. Specifically, in traditional corn-based fermented beverages, a great diversity of lactic acid bacteria (LAB), molds, and yeasts have been found to utilize corn nutrients, improving the nutritional properties of the product. For example, LAB increases the free amino acids content, B complex vitamin, minerals (calcium, iron, and zinc), volatile compounds that contribute to the organoleptic characteristics of maize-based fermented beverages [3].

*Tejuino* is a traditional non-distilled fermented beverage from Mexican produced with germinated or nixtamalized maize. Currently, this beverage is produced and consumed mainly in the western and north-western states of Mexico (*e.g*., Nayarit, Jalisco, Zacatecas, Sinaloa, Sonora, and Chihuahua) [4]. This beverage has an important traditional ceremonial use in ethnic groups. Its production has spread within the indigenous communities. Nevertheless, *Tejuino* is part of the gastronomy of traditional beverages of Mexico which can be found in urban regions of Nayarit [5].

During the production of *Tejuino*, the temperature, types of microorganisms, pH, production zone, and fermentation time are variables that determine the nutritional and microbiological composition [6]. The fermentation process for *Tejuino* is spontaneous and uncontrolled, principally for the artisanal kind; therefore, the sensory characteristics and quality are important variables due to several microorganism types being involved, such as the *Saccharomyces* genus and LAB, principally [7]. These microorganisms contribute to the flavor development through a carbohydrate metabolism, proteolysis, and amino acid catabolism, as well lipolysis and a fatty acids metabolism [4,5,8]. In this sense, some microorganisms have been identified in traditional beverages produced from corn, such as *Saccharomyces cerevisiae*, *Candida inconspicua*, amylolytic LAB (*Zymomona mobilis*), and LAB (*Lactobacillus plantarum*, *Lactococcus lactis*, *Lactobacillus acidophilus,* and *Lactobacillus casei*), which are involved in the flavor development [9].

Regarding the flavor, it is one of the most important sensory attributes of traditional fermented beverages. The taste of these beverages is determined by the presence of volatile compounds obtained by the metabolism of carbohydrates, proteins, lipids, free amino acids, and microbiota action [10,11]. However, the information available on these properties for *Tejuino* beverage has not been reported for the last 20 years. This study aims to analyze the chemical and microbial composition and characterization of volatile compounds from artisanal and commercial *Tejuino* beverages.

## 2. Materials and Methods

### 2.1. Collection of Samples

Samples of commercial *Tejuino* were purchased at local mobile stores and artisanal *Tejuino* from Wixárika ethnic groups from Nayarit, Mexico. Samples were collected in two seasons (winter and spring) in different regions of Nayarit. Samples from artisanal *Tejuino* were codified as “AR” (El Nayar), “AY” (La Yesca), “AW” (Wirikuta), and “TZ” (Zitacua); commercial *Tejuino* samples were codified as “TC” (City center), “TL” (Leon Street), “TM” (Plaza de la Música), and “TT” (Tecnológico Zone), obtaining a total of eight different beverages. All samples were transported under refrigeration conditions to the laboratory for further analysis.

A fresh sample (1 L) was used for physicochemical analysis (pH, acidity, moisture, total solids) of volatile compounds; for the microbiological analysis, 200 mL of sample was aseptically obtained and placed in sterile flasks and immediately analyzed. On the other hand, 800 mL was frozen at −80 °C and lyophilized (FreeZone 6, Labconco, Kansas City, USA) to determine fat, protein, and ash.

### 2.2. Physicochemical Analysis

Physicochemical parameters of *Tejuino* were determinate according to standard methodologies [12]; total solids, moisture by difference, were determined by the oven drying method (method 925.23), protein (micro-Kjeldahl method 991.20), fat (Soxhlet method: 989.04), ash (gravimetric method: 990.19), and acidity (method: 920.12). Concentration of carbohydrates was determinate by difference: (100—(total solids + protein + fat + ashes)) [13]. Furthermore, pH was recorded using a Microprocessor pH meter (Hanna Instruments, pH 211, Woonsocket, RI, USA).

### 2.3. Microbiological Analysis

The microbiology quality was determined following the methodology reported by Torres-Llanez et al. [14]. For this, 10 mL of each *Tejuino* sample was mixed with 90 mL of peptone water at room temperature and series of 10-fold dilutions were performed. Total Coliform Bacteria (TCB) was evaluated by the most probable number (MPN) method in Lauryl Sulfate Broth (BD Difco, Sparks, MD, USA), and incubated at 25 °C for 48 h. Aerobic mesophilic bacteria (AMB) in Plate Count Agar (BD Difco) at 35 °C for 48 h; molds and yeasts (MY) in Potato Dextrose Agar (BD Difco) acidified at pH 3.5 with tartaric acid solution (10% *w/v*) (FAGALAB, Sinaloa, Mexico) at 25 °C for 5 days. The cell count was reported as log_10_ colony-forming units (CFU)/mL. All analyses were performed following the specifications of the Official Mexican Standard (NOM) [15,16,17].

On the other hand, LAB concentration of *Streptococcus* spp and *Lactococcus* spp was determined in M17 agar (BD Difco) supplemented with lactose and dextrose (5%) and incubated at 42 °C and 30 °C for 48 h, respectively; *Lactobacillus* spp. in de Man, Rogosa and Sharpe agar (MRS BD Difco) at 37 °C for 48 h under anaerobic conditions (BD GasPak Anaerobe Container System, MD, USA). The cell concentration was reported as log_10_ CFU/mL.

### 2.4. Isolation of Lactic Acid Bacteria and Yeast

A minimum of two representative LAB colonies was obtained for each medium selected (M17 and MRS agar). Three culture steps in agar–broth–agar were performed, and colony morphology characteristics such as shape, margin, and elevation were considered [18]. Later, the bacteria and yeasts were grown in a specific medium for 18 h. A catalase and oxidase test by Kovacs method and Gram staining were performed. The isolated strains were visualized by microscopy (100 × objective, Modelo AXIO, ZEISS Corporation, Germany). Finally, the strains were stored at −80 °C using 80% glycerol (*v*/*v*) [19].

### 2.5. Identification of LAB and Yeasts Strains

LAB identification; each strain was cultured for 18 h in MRS (anaerobic conditions), M17 broth at 37 °C, 30 °C, and 40 °C. Then, 1 mL of each strain was centrifuged (16,000× *g*, 10 min) (Spectrafuge 16M, Labnet, Woodbridge, NJ, USA) to obtain a pellet and the DNA extraction was performed using the commercial protocol kit PrepMan Ultra Sample Preparation Reagent (Applied Biosystems, Foster City, CA, USA). The DNA purity was determinate at 260 nm (NanoDrop 2000c UV–Vis, Thermo Fisher Scientific, Wilmington, DE, USA), and the DNA integrity was evaluated in agarose gel (Sigma-Aldrich, MO, USA) electrophoresis (80 V, 30 min). It was visualized in a photodocumenter (Gel DocTM XR+System, Bio-Rad, CA, USA). The analysis of the 16S rRNA method was performed following the protocol of MicroSEQ^TM^ 500 identification Systems (Applied Biosystems). The PCR conditions for amplification was performed in a thermocycler endpoint (Eppendorf, Hamburg, Germany). The PCR conditions for denaturation were at at 95 °C for 10 min, 30 cycles of 95 °C for 30 s, 60 °C for 30 s, and 72 °C for 45 s, final extension of 72 °C for 10 min and final step at 4 °C. For the sequencing, 25 cycles were performed at 96 °C for 10 s, 50 °C for 5 s, and 60 °C for 4 min.

For the identification of yeast, this was grown in PDA broth at 25 °C, 18 h. Subsequently, 1 mL of culture was centrifuged (16,000× *g*, 10 min) (Spectrafuge 16M) for the extraction of DNA using the commercial protocol Wizard Genomic DNA Purification Kit (Promega, Madison, WI, USA). The amplification method was based on the MicroSEQ^TM^ D2 rDNA Fungal Sequencing Kit (Applied Biosystems). The PCR conditions for amplification initial step was at 95 °C for 10 min, 35 cycles at 95 °C for 30 s, 53 °C for 30 s, 72 °C for 1 min, final extension 72 °C for 10 min. For the sequencing run, 25 cycles were performed at 96 °C for 10 s, 50 °C for 5 s, and 60 °C for 4 min.

Each PCR product was purified with ExoSAP-IT^TM^ PCR Product Cleanup Reagent (Affymetrix, Santa Clara, CA, USA) and, then, sequencing reactions were purified using the BigDye Xterminator^TM^ purification kit (Applied Biosystems) and, the purified sequencing reactions were examined in a 3500 Genetic Analyzer (Applied Biosystems). The sequences obtained were analyzed in the MicroSEQ software v2.0 (Applied Biosystems) and compared with the library of GenBank database (Accessed 1 January 2021; http://www.ncbi.nlm.nih.gov/BLAST/) using the Blast program of NCBI to determinate the identity of the strains. Sequences with a percentage identity > 99% were considered to belong to the same species.

### 2.6. Analysis of Volatile Compounds by Solid-Phase Microextraction Gas Chromatography Mass Spectrometry (SPME-GC-MS)

Headspace solid-phase microextraction and gas chromatography (HS-SPME-GC) method were used following the methodology reported by Reyes-Díaz et al. [20]. For this, fresh *Tejuino* samples (5 mL), 2 g of NaCl, and 5 μL of dodecanoic acid methyl ester (internal standard, 1000 ppm in methanol, PolyScience C., Niles, IL, USA) were placed in screw-capped glass vials (20 mL, 22.75 × 75 mm) and sealed with headspace vial caps (18 mm magnetic PTFE/Sil, Agilent Technologies, Basel, Switzerland). Then, vials were placed on the GC Sampler system (GC autosampler 80, Agilent Technologies). Before SPME extraction, samples were allowed to equilibrate at 70 °C (150 rpm, 30 min). Afterward, a 75 μm CAR-PDMS fiber (Supelco Co., Bellefonte, PA, USA) was exposed to the sample headspace while shaking (150 rpm) at 70 °C for 60 min. Then, the fiber was withdrawn into the needle of the SPME device and immediately desorbed at 250 °C for 10 min in splitless mode. Volatile compounds were determined into GC-MS System (Agilent Technologies 7890A/5975C, Santa Clara, CA, USA), and the injector was provided with an inlet liner (78.5 mm × 6.5 mm O.D. × 0.75 mm I.D., Sigma-Aldrich, St. Louis, MO, USA). Helium was used as the carrier gas at 1.5 mL/min.

The compounds were separated by a high-polarity DB-WAX column (60 m length, 0.25 mm I.D., 0.25-μm film thickness, Agilent J&W Scientific) with a carrier gas at 1 mL/min. The initial oven temperature was held at 45 °C for 12.5 min, increased to 114 °C (rate of 4 °C/min for 6 min), then to 143 °C (rate of 7 °C/min for 15 min), and, finally, increased to 165 °C (rate of 15 °C/min, for 15 min). Volatile compounds were tentatively identified by comparing their spectra to those from the National Institute of Standards and Technology MS library (Soft. v. 2.0 g., 2011).

### 2.7. Statistical Analysis

All dates were analyzed using one way-ANOVA, ant the differences among samples were compared using the Fisher LSD test with *p* < 0.05. Principal component analysis (PCA) by factor loadings and factor scores was performed to obtain volatile compounds data; clusters were determined by k-means clustering analysis. All the statistical analyses were performed by using the NCSS-2007 statistical software (Kaysville, UT, USA).

## 3. Results and Discussion

### 3.1. Chemical Composition

The results of the physicochemical composition of the samples of *Tejuino* are shown in Table 1. Overall, the parameters evaluated showed differences (*p* < 0.05) among the samples evaluated. In particular, the artisanal samples presented a higher moisture (89.73–92.66%) compared to the commercial samples (83.35–88.29%) and, therefore, the TS were lower for the artisan samples (7.34 to 10.28%). The protein content showed a difference (*p* < 0.05), being 7.3-fold higher for the artisanal samples than commercial. On the other hand, the fat content was 1.81-fold and ash was 3.10-fold higher for artisanal samples (*p* < 0.05). On the contrary, the CHOS content was higher for commercial samples with a mean of 78.70%; meanwhile, for artisanal samples, it was 62.48% (*p* < 0.05). The pH range was 2.91 to 3.52 and 3.36 to 3.96; for acidity there was 0.4 to 1.22 and 0.27 to 0.57 meq of lactic acid/L for the artisanal and commercial beverages, respectively, the artisanal beverages being the ones to show the higher acidity. Although, a difference of (*p* > 0.05) among artisanal and commercial *Tejuino* was seen.

The elaboration process could explain the difference in the physicochemical composition between artisanal and commercial *Tejuino* beverages. Corn germination is conducted in dark conditions; then, the sun drying process proceeds (2–3 days), finally being mixed with water. The liquid was cooking and the sediments were fermented for 24 h at room temperature. Afterwards, both samples were mixed, filtered, and the filtered sample was left to ferment for 7 days. On the other hand, commercial *Tejuino* is produced from nixtamalized corn, followed by the addition of piloncillo to initiate fermentation for 12–24 h [5]. In particular, the combined processes of fermentation and germination improve the nutritional compositions of beverages. A study reported by Hiran et al. [21] showed an enhanced—up to two-fold—protein content of fermented germinated maize seeds by LAB. Furthermore, during germination, endogenous enzymes hydrolyze the maize compounds, e.g., prolamins and glutelins [22].

The CHOS concentration for commercial *Tejuino* may be influenced by the addition of “piloncillo” (brown sugar, prepared from the undistilled juice of sugar that contains >70% of sugar). On the contrary, the starch present in the germinated corn is hydrolyzed into sugars such as glucose and saccharose [23]. During the fermentation, the microorganisms reduce the carbohydrates content up to 20% [21], as well as the ash content, but enhance the protein content [24].

The values of pH and acidity for artisanal and commercial *Tejuino* did not show differences, but AR and AY were the lowest. Fermentation is spontaneous and uncontrolled in traditional fermented products; artisanal *Tejuino* has up to 6 days of fermentation, where several microorganisms and enzymatic processes hydrolyze molecules such as lipids, proteins, and carbohydrates. For instance, Ben Omar and Ampe, [25] associated the pH reduction due to organic products (e.g., lactic acid, formic acid) and ethanol by the action of microorganisms. Although the fermentation time for commercial *Tejuino* is only 12 to 24 h, the “*Tejuino viejo*” (starter culture unless 24 h of fermentation), and, finally, some additives are incorporate, such as salt and lemon juice, which may influence the pH values.

### 3.2. Microbial Quality

The microbial quality of *Tejuino* beverages is shown in Table 2. Overall, the artisanal samples showed the highest concentrations for all types of microorganisms evaluated. Only total coliform bacteria were absent in all samples. All cell concentrations were different (*p* < 0.05) among samples, but higher for artisanal beverages. The MY range was 7.32 to 8.47 and 4.74 to 7.93 log10 CFU/mL; AMB was 7.27 to 8.66 and 7.17 to 7.59 93 log_10_ CFU/mL for artisanal and commercial beverages, respectively.

The physicochemical composition of *Tejuino* determines the microbial diversity. Some conditions of low pH may be a factor that affects the presence of some microorganisms. For instance, LAB predominates in an acidic environment, but there was less or an absence of total enterobacteria [26]. On the contrary, LAB were the predominant group in these beverages. During the nixtamalization processes, the concentration of carbohydrates was reduced, starch being the main carbohydrate available for LAB [27]. This could explain why the commercial beverage had a lower concentration of LAB. Regarding artisanal *Tejuino,* it showed the highest concentration. This great diversity in the concentration of microorganisms has been reported in other traditional maize-based beverages, such as *Pozol* [25,27], *Atole Agrio* [28], and commercial *Tejuino* [29].

The microbiological quality for both beverages showed marked differences in concentration, which could be attributed to the fact that both processes are non-standardized methods, indicating an absence of good manufacturing practices. However, the presence of microorganisms determines the sensory characteristics of this type of beverage, and even the possible health effects attributed to the great diversity of metabolites present, and even the microbiota present have been documented in other beverages [30]. Moreover, during spontaneous fermentation, bacteria and yeasts produce several metabolites available for other microorganisms, showing a microbial diversity [31].

### 3.3. Identification of LAB and Yeast Strains

LAB and yeast were identified molecularly by comparing the16S rRNA gene sequences with NCBI sequences available in this database (Table 3). Twenty-eight strains were identified with a 100% similarity except for strain number twenty-eight, which showed 95%. The main yeasts identified were *Pichia occidentalis* and *Pichia kudriavzevii* for commercial *Tejuino*, and *Saccharomyces cerevisiae* for the artisanal *Tejuino* samples. On the other hand, LAB identification, the predominant bacteria identified were *Limosilactobacillus fermentum* (five strains) and *Lactiplantibacillus plantarum* (three strains) for commercial and artisanal *Tejuino*, *Enterococcus durans* (five strains) for commercial *Tejuino* and two strains for artisanal *Tejuino*, *Enterococcus faecium* and *Staphylococcus warneri* (two strains) for commercial, and *Lactococcus lactis* for artisanal *Tejuino.*

In this sense, important characteristics were documented for some identified microorganisms, which could be of great interest for future studies. In the yeast group, *Pichia* genus has been isolated from *Chicha* (indigenous Andean beer) [32], and has been reported as a start culture [33]. *Pichia kudriavzevii* has been characterized by the production of biofilms at a low pH [34]; *Pichia occidentalis,* with the potential to produce phytases and bioethanol in prolonged fermentation times [35,36], also showed a stress tolerance [32]. This yeast has been reported in other fermented beverages such as cachaça, tequila, mezcal, wine, and beer [37]. *Saccharomyces cerevisiae* has been widely reported for its fermentation properties and ethanol production [32,38], and is present in several traditional fermented beverages of Mexico such as *Atole agrio* [39], *Tesgüino* [6], and *Tepache* [40].

LAB are the dominant microorganism in several foods, but the existence of consortium helps to promote the diversity of microorganisms; LAB produces organic acids that decrease pH and promote the growth of yeast [41]. Furthermore, LAB contributes to food preservation and flavor development by organic acid production. Some reports show increasing digestibility in vitro of the proteins and starch of maize due to LAB consortium by fermentation [42]. However, we found undesirable bacteria such as *Staphylococcus*, related to food handling practices [43].

During spontaneous fermentation, bacteria and yeasts release several metabolites and promote the growth of other microorganisms. Thus, this interaction enhances the nutritional, rheological, and sensory properties of food [31]. This effect has been documented for other traditional beverages [6,39,40].

### 3.4. Volatile Compounds

The volatile compounds found in the eight samples of artisanal and commercial *Tejuino* beverages are shown in Table 4. Eighty-nine volatile compounds were identified; however, only ten compounds were detected in all beverages (decanoic acid ethyl ester, acetic acid 2-phenylethyl ester, hexadecanoic acid ethyl ester, ethanol, 1-pentanol, phenylethyl alcohol, octanoic acid, benzaldehyde, creosol, and phenol, 4-ethyl) and showed a difference among artisanal and commercial beverages (*p* < 0.05) except for cresol and phenol 4-ethyl. In this regard, esters, benzenes, and aldehydes were the highest number of volatile compounds identified (26, 17 and 12 compound, respectively). Our data showed the highest relative abundance of volatile compounds for artisanal samples, the esters group being the predominant compounds. The “AR” beverage showed the highest esters; meanwhile, “AW” was for aldehydes and benzenes. Esters may provide fruity and floral flavors in some food; thus, decreasing the astringent aroma of fatty acids and amines [44].

Although alcohols were not the predominant group, it is important to highlight that ethanol showed the highest concentration, mainly for artisanal beverages, with 4.17, 3.01, 4.40, and 4.21 ppm, followed by phenyl ethyl alcohol with 1.13, 2.01, 1.6, and 0.9 ppm for “AR”, “AW”, “AY” and “AZ”, respectively. In this sense, the higher alcohol concentration in the artisanal sample could be attributed to the presence of *Saccharomyces cerevisiae*. This yeast produces enzymes such as amylases, lipases, esterases, and phytases which promote flavor and aroma, and are the most employed for ethanol production, ketones, aldehydes, and esters. Furthermore, the high LAB concentration may favor the enzymes production for hydrolyzing corn starch and the yeasts used for their metabolism, which release vitamins and soluble nitrogen for LAB growth [45]. Other cereal-based fermented co-cultures of LAB and yeast showed a higher alcohol content [46].

Other compounds such as methanesulfonic anhydride, furan 2-penty, ethenone 1-(2-furanyl), and cyclopropane pentyl were in both types of *Tejuino*; meanwhile, 2-vinylfuran, methyl vinyl ketone, cyclopentane methyl, hexadecane, and bicycle(6.1.0)nonane, 9-(1-mehtylethylidene) were found only in artisanal samples. For germinated maize, the volatile compounds such as lactic acid, 2,3-butanediol, dodecane, benzofurans, and tridecane are produced by microorganisms such as *Lactobacillus plantarum* and *Lactococcus lactis* [21].

**Figure 1:**
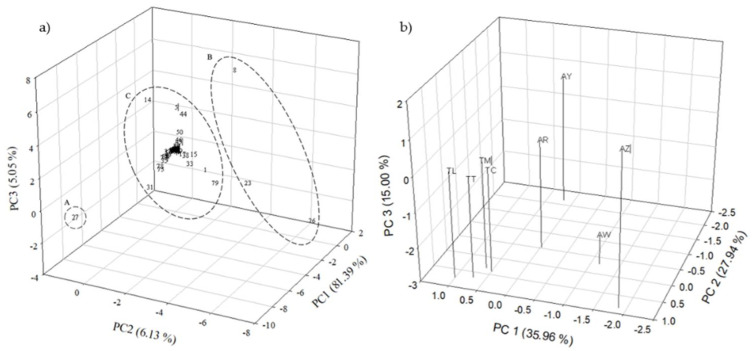
Principal component analysis (PCA). (**a**) PCA of the first three factor scores and factor loadings of volatile compounds of all samples of *Tejuino* fresh artisanal and commercial samples. (**b**) PCA of the three first factor loadings of samples of *Tejuino*. AR: El Nayar; AW: Wirikuta; AY: La Yesca; AZ: Zitacua; TC: Tepic Center; TL: León Street; TM: Music square; TT: Tecnólogico zone.

Additionally, the analysis of the PCA showed the clustering of volatile compounds and *Tejuino* sample (Figure 1). Figure 1a shows the PCA of 89 identified compounds, and three main groups were identified by the k-means cluster analysis and explained with 81.39%, 6.13%, and 5.05% for PC1, PC2, and PC3, respectively. In cluster A found showed the highest concentration of volatile compounds and ethanol was included. In cluster B are compounds that did not show a significant difference; cluster C only contained tree compounds (decanoic acid ethyl ester, hexadecenoic acid ethyl ester, and (E)-9-octadecenoic acid ethyl ester).

The PCA for *Tejuino* samples is shown in Figure 1b. This analysis showed three components that explained the 35.96%, 27.94%, and 15.00% for PC1, PC2, and PC3, respectively. Commercial beverages (TC, TL, TM, and TT) were grouped at the same cluster. However, artisanal beverages (AR, AW, AY, and AZ) showed a high dispersion, indicating a higher variability among them, considering the distribution of the identified volatile compounds.

Volatile compounds such as esters derived from fatty acids detected in this study could promote fruity notes and have been related to the presence of yeast strains such as *Saccharomyces cerevisiae* [47,48,49]. Thus, the esters found in *Tejuino* samples could be attributed to their content of alcohols, which are precursors of these compounds [50].

On the other hand, the content of ethanol was associated with *Saccharomyces cerevisiae*, which is the most commonly employed yeast for ethanol production [51,52], and this was identified for the artisanal samples of our study. Moreover, yeast produces long-chain and complex alcohols that, together with esters, have an interesting flavor, such as phenylethanol, which possesses a rose-like aroma [53,54], as well as sweet alcohol, rough, and fruity aromas [55].

On the other hand, D-limonene (terpene) was reported in both samples and has been associated with a lemon-like [56], flowery, and lilac odor [57,58]. The compounds and β-myrcene were reported in others beverages composed of maize [58]. Otherwise, aldehydes were found in both types of *Tejuino.* For instance, hexanal has been identified in other cereal-based fermented products and sorghum malt beverages, and has been reported having strong antimicrobial properties against pathogenic microorganisms at low concentrations [59,60].

The relative abundance for aldehydes, only for artisanal *Tejuino,* may be attributed to soaking that disrupt the physical structure to release volatile compounds and enhance lipoxygenase activity [61]. The fermentative changes directly influence final fermented physical properties, chemical, biological, and sensory properties. This activity is associated with microorganisms, which generate various volatile compounds that promote the development of flavors in fermented foods [62].

## 4. Conclusions

Their production process primarily determined the physicochemical characteristics and microbiological quality of the *Tejuino* beverages. In this aspect, the artisanal beverage was outstanding, and showed a great diversity of volatile compounds. The isolated microorganisms identified were yeasts and lactic acid bacteria, which may be considered for future studies to evaluate the potential bioactivity for their use in industrial processes, as well as an examination of microbiota, which could be carried out to assess its possible potential use in the production of compounds with a biotechnological application.

This study allowed us to know and update the available information on this important traditional fermented beverage of Mexico.

## Figures and Tables

**Table 1:** Physicochemical composition of artisanal and commercial *Tejuino* beverages from Nayarit, Mexico.

	Artisanal *Tejuino*				Commercial *Tejuino*			
Item	AR	AW	AY	AZ	TC	TL	TM	TT
^1^ Moisture	89.8 ± 0.22 ^bc^	92.66 ± 0.34 ^d^	89.73 ± 0.07 ^bc^	91.66 ± 1.20 ^cd^	87.70 ± 1.01 ^b^	84.65 ± 0.32 ^a^	88.29 ± 0.02 ^b^	83.35 ± 3.77 ^a^
^1^ TS	10.2 ± 0.22 ^bc^	7.34 ± 0.34 ^a^	10.28 ± 0.07 ^bc^	8.34 ± 1.20 ^ab^	12.30 ± 1.01 ^c^	15.36 ± 0.33 ^d^	11.72 ± 0.02 ^c^	16.65 ± 3.77 ^d^
^1^ Protein	18.60 ± 0.92 ^f^	27.05 ± 0.32 ^g^	16.50 ± 0.12 ^e^	6.17 ± 0.08 ^d^	2.84 ± 0.14 ^bc^	2.32 ± 0.25 ^b^	3.00 ± 0.25 ^c^	1.23 ± 0.01 ^a^
^1^ Fat	7.67 ± 0.00 ^d^	9.01 ± 0.58 ^d^	5.67 ± 0.00 ^c^	4.85 ± 0.38 ^c^	4.72 ± 0.04 ^b^	4.23 ± 0.00 ^b^	4.02 ± 0.48 ^b^	2.04 ± 0.00 ^a^
^1^ CHOS *	59.42 ± 1.25 ^d^	49.35 ± 0.10 ^e^	63.62 ± 0.14 ^c^	77.53 ± 1.23 ^ab^	79.27 ± 1.24 ^ab^	76.70 ± 0.97 ^b^	79.32 ± 0.69 ^ab^	79.52 ± 3.77 ^a^
^1^ Ash	3.78 ± 0.57 ^d^	7.24 ±0.02 ^e^	4.01 ± 0.13 ^d^	2.73 ± 0.08 ^c^	0.87 ± 0.06 ^a^	2.39 ± 0.04 ^c^	1.93 ± 0.01 ^b^	0.56 ± 0.01 ^a^
pH	2.91 ± 0.01 ^a^	3.52 ± 0.01 ^c^	2.91 ± 0.01 ^a^	3.48 ± 0.01 ^b^	3.63 ± 0.01 ^d^	3.96 ± 0.01 ^e^	3.52 ± 0.01 ^c^	3.36 ± 0.01 ^b^
Acidity ^‡^	0.4 ± 0.01 ^b^	0.76 ± 0.01 ^f^	0.68 ± 0.01 ^e^	1.22 ± 0.01 ^g^	0.27 ± 0.02 ^a^	0.57 ± 0.03 ^d^	0.55 ± 0.02 ^d^	0.47 ± 0.01 ^c^

Values show the means ± standard deviation (*n* = 3); ^1^ Values are expressed as percentage. Different letters for the same row indicate significant differences (*p* ≤ 0.05) among samples. AR: El Nayar; AW: Wirikuta; AY: La Yesca; and AZ: Zitacua; TC: Tepic Center; TL: León Street; TM: Music square; TT: Tecnólogico zone; TS: total solids; CHOS *: carbohydrates, ^‡^ meq LA/mL.

**Table 2:** Microbiological quality of artisanal and commercial *Tejuino* beverages from Nayarit, Mexico.

	Artisanal *Tejuino*				Commercial *Tejuino*			
Microorganisms	AR	AW	AY	AZ	TC	TL	TM	TT
MY	8.33 ± 0.01 ^g^	8.23 ± 0.05 ^f^	8.47 ± 0.01 ^h^	7.32 ± 0.02 ^d^	7.26 ± 0.04 ^c^	6.76 ± 0.02 ^b^	7.93 ± 0.03 ^e^	4.74 ± 0.04 ^a^
44AMB	8.66 ± 0.01 ^e^	8.23 ± 0.01 ^c^	8.40 ± 0.01 ^d^	7.27 ± 0.02 ^a^	7.15 ± 0.01 ^a^	7.11 ± 0.38 ^a^	7.59 ± 0.05 ^b^	7.17 ± 0.06 ^a^
TCB *	<3	<3	<3	<3	<3	<3	<3	<3
Lactic Acid Bacteria								
*Lactobacillus* spp.	8.28 ± 0.02 ^c^	8.13 ± 0.03 ^c^	8.33 ± 0.01 ^c^	6.98 ± 0.01 ^a^	7.18 ± 0.03 ^a^	7.14 ± 0.33 ^a^	7.64 ± 0.01 ^b^	7.13 ± 0.01^a^
*Lactococcus* spp.	8.46 ± 0.10 ^b^	8.22 ± 0.01 ^b^	8.47 ± 0.01 ^b^	7.19 ± 0.05 ^a^	7.28 ± 0.03 ^a^	6.86 ± 0.68 ^a^	7.42 ± 0.02 ^a^	7.15 ± 0.07 ^a^
*Streptococcus* spp.	8.11 ± 0.01 ^d^	8.18 ± 0.05 ^d^	8.46 ± 0.10 ^e^	7.18 ± 0.01 ^bc^	7.26 ± 0.01 ^bc^	6.87 ± 0.80 ^ab^	6.89 ± 0.11 ^b^	7.15 ± 0.07 ^bc^

Values represent the means ± standard deviation (*n* = 3). Different letters for the same microorganisms group indicate significant differences (*p* ≤ 0.05) among samples. AR: El Nayar; AW: Wirikuta; AY: La Yesca; AZ: Zitacua; TC: Tepic Center; TL: León Street; TM: Music square; TT: Tecnólogico zone. Total count of molds and yeasts (MY), aerobic mesophilic bacteria (AMB), and total coliform bacteria (TCB). * MPN / mL.

**Table 3:** Phylogenetic identification results of isolated yeasts and bacteria strains of commercial and artisanal *Tejuino*.

Commercial *Tejuino*			Artisanal *Tejuino*		
Strain	Identification	Accession	Strain	Identification	Accession
1	*Pichia occidentalis*	MN904761.1	4	*Saccharomyces cerevisiae*	MT649488.1
2	*Pichia kudriavzevii*	JF715184.1	5	*Saccharomyces cesevisiae*	
3	*Pichia kudriavzevii*		6	*Saccharomyces cerevisiae*	
7	*Acetobacter orientalis*	MT416429.1	11	*Lactococcus lactis*	MT645510.1
9	*Enterococcus faecium*	MK332450.1	17	*Lactiplantibacillus plantarum*	CP050805.1
12	*Limosilactobacillus fermentum*		18	*Lactiplantibacillus plantarum*	
13	*Limosilactobacillus fermentum*	MT613608.1	20	*Lactiplantibacillus plantarum*	
14	*Limosilactobacillus fermentum*		10	*Enterococcus durans*	MT604840.1
15	*Limosilactobacillus fermentum*		27	*Enterococcus durans*	
16	*Limosilactobacillus fermentum*		28	*Enterococcus hirae*	KX752853.1
21	*Staphylococcus warneri*	MT642942.1			
22	*Staphylococcus warneri*				
8 23	*Enterococcus durans* *Enterococcus durans*	MT604840.1			
24	*Enterococcus durans*				
25	*Enterococcus durans*				
26	*Enterococcus durans*				

**Table 4:** Volatile compounds identified in *Tejuino* beverage by headspace solid-phase microextraction and GC-MS (ppm).

			Artisanal *Tejuino*				Commercial *Tejuino*		
No.	Volatile Compound	RT	AR	AW	AY	TC	TL	TM	TT
**Esters**									
1	Ethyl acetate	6.01	0.104 ± 0.06 ^a^	0.081 ± 0.05 ^a^	1.041 ± 1.29 ^a^	ND	0.008 ± 0.01 ^a^	0.049 ± 0.02 ^a^	0.008 ± 0.01 ^a^
2	Butanoic acid, ethyl ester	10.2	ND	ND	ND	ND	ND	ND	0.010 ± 0.00 ^a^
3	Pentanoic acid, 3-methyl-, ethyl ester	21.36	0.008 **±** 0.01 ^a^	ND	0.021 ± 0.00 ^a^	ND	ND	ND	ND
4	Propanoic acid, 2-hydroxy-, ethyl ester, (S)	26.4	ND	ND	0.005 ± 0.00 ^a^	ND	ND	ND	ND
5	Octanoic acid, ethyl ester	30.76	0.42 ± 0.18 ^a^	0.139 ± 0.07 ^a^	ND	0.907 ± 0.77 ^a^	0.381 ± 0.4 1 ^a^	0.019 ± 0.02 ^a^	0.005 ± 0.00 ^a^
6	Nonanoic acid, ethyl ester	34.86	0.026 ± 0.01 ^a^	0.0372 ± 0.05^a^	ND	0.015 ± 0.01^a^	ND	ND	DN
7	Decanoic acid, methyl ester	37.47	ND	ND	ND	ND	ND	ND	DN
8	Decanoic acid, ethyl ester *	39.19	0.998 ± 0.4 ^d^	0.046 ± 0.02 ^abc^	0.875 ± 0.06 ^cd^	0.252 ± 0.14 ^a^	0.753 ± 0.61 ^bcd^	0.035 ± 0.02 ^ab^	0.008 ± 0.00 ^bcd^
9	Octanoic acid, 3-methylbutyl ester	39.82	0.029 ± 0.01	ND	ND	ND	ND	ND	ND
10	Butanedioic acid, diethyl ester	40.58	0.005 ± 0.00 ^a^	ND	ND	ND	0.015 ± 0.00 ^a^	ND	ND
11	Ethyl 9-decenoate	40.68	0.027 ± 0.01 ^a^	ND	ND	0.044 ± 0.02 ^a^	ND	0.004 ± 0.00 ^a^	ND
12	Undecanoic acid, methyl ester	40.82	ND	ND	ND	ND	ND	ND	ND
13	Benzeneacetic acid, ethyl ester	44.12	0.008 ± 0.00 ^a^	0.024 ± 0.01 ^a^	0.023 ± 0.00 ^a^	ND	ND	ND	ND
14	Acetic acid, 2-phenylethyl ester *	45.45	0.107 ± 0.11 ^a^	0.175 ± 0.07 ^a^	0.491 ± 0.05 ^a^	0.148 ± 0.18 ^a^	0.349 ± 0.02 ^a^	0.998 ± 0.52 ^b^	0.043 ± 0.03 ^ab^
15	Dodecanoic acid, ethyl ester	46.84	0.430 ± 0.15 ^ab^	0.052 ± 0.02 ^ab^	0.516 ± 0.03 ^ab^	0.017 ± 0.01 ^a^	0.056 ± 0.01 ^b^	0.01 ± 0.01 ^b^	0.007 ± 0.00 ^b^
16	Pentanoic acid, 3-methylbutyl ester	48.25	0.017 ± 0.01 ^a^	ND	0.027 ± 0.02^a^	ND	ND	ND	ND
17	Benzenepropanoic acid, ethyl ester	48.83	0.040 ± 0.02 ^a^	ND	0.218 ± 0.05^a^	ND	ND	ND	ND
18	2(3H)-Furanone, dihydro-5-pentyl	58.3	0.077 ± 0.04 ^a^	0.382 ± 0.14 ^a^	ND	0.31 ± 0.17 ^a^	ND	ND	ND
19	Nonanoic acid, 9-oxo-, ethyl ester	61.81	0.003 ± 0.00 ^a^	ND	0.002 ± 0.00 ^a^	ND	ND	ND	ND
20	Diethyl suberate	62.47	0.019 ± 0.01	ND	ND	ND	ND	ND	ND
21	Pentadecanoic acid, ethyl ester	63.97	0.004 ± 0.00 ^a^	ND	0.025 ± 0.00 ^a^	ND	ND	ND	ND
22	Hexadecanoic acid, methyl ester	68.38	0.008 ± 0.002 ^a^	ND	ND	ND	ND	ND	ND
23	Hexadecanoic acid, ethyl ester *	71.83	0.28 ± 0.15 ^a^	0.082 ± 0.02 ^a^	2.19 ± 0.36 ^b^	0.016 ± 0.01 ^a^	0.121 ± 0.02 ^a^	0.022 ± 0.00 ^a^	0.059 ± 0.04 ^a^
24	Decanedioic acid, diethyl ester	78.89	0.043 ± 0.02	ND	ND	ND	ND	ND	ND
25	8-Nonenoic acid, ethyl ester	81.85	0.016 ± 0.01	ND	ND	ND	ND	ND	ND
26	(E)-9-Octadecenoic acid ethyl ester	98.55	0.298 ± 0.23 ^a^	ND	3.93 ± 0.69 ^b^	ND	0.098 ± 0.02 ^a^	ND	0.022 ± 0.01 ^a^
**Alcohols**									
27	Ethanol*	7.06	4.169 ± 1.56 ^b^	3.095 ± 1.72 ^ab^	4.404 ± 0.36 ^b^	4.21± 3.06 ^b^	0.9 ± 0.52 ^a^	1.67 ± 0.75 ^a^	1.038 ± 0.45 ^a^
28	1-Pentanol*	20.34	0.101 ± 0.04 ^a^	0.135 ± 0.06 ^ª^	0.15 ± 0.01 ^a^	0.199 ± 0.18 ^a^	0.036 ± 0.01 ^a^	0.033 ± 0.02 ^a^	0.022 ± 0.01 ^a^
29	2,6-Octadien-1-ol,3,7-dimethyl-,acetate, (Z)	41.62	ND	ND	ND	ND	0.029 ± 0.00 ^a^	0.001 ± 0.00 ^a^	0.009 ± 0.00 ^b^
30	Benzyl alcohol	48.24	ND	0.006 ± 0.00	ND	ND	ND	ND	ND
31	Phenylethyl Alcohol *	50.69	1.125 ± 0.43 ^cd^	2.096 ± 0.78 ^e^	1.597 ± 0.18 ^de^	0.901 ± 0.7 ^bc^	0.322 ± 0.06 ^ab^	0.225 ± 0.12 ^ab^	0.16 ± 0.01 ^a^
**Organic acids**									
32	Alpha-pyrone-6-carboxylic acid	28.95	ND	ND	ND	0.035 ± 0.03^a^	ND	0.003 ± 0.00^a^	ND
33	Acetic acid	30.2	ND	0.022 ± 0.02 ^a^	0.557 ± 0.04 ^a^	ND	ND	0.003 ± 0.00 ^a^	0.067 ± 0.04 ^a^
34	Butanoic acid	38.06	ND	ND	ND	ND	ND	ND	0.183 ± 0.04 ^a^
35	Octanoic acid	58.85	0.161 ± 0.07 ^a^	0.118 ± 0.12 ^a^	0.32 ± 0.04 ^a^	1.264 ± 0.92 ^b^	0.019 ± 0.02 ^a^	0.072 ± 0.08 ^a^	0.02 ± 0.00 ^a^
36	Sorbic Acid	63.57	ND	ND	ND	ND	ND	ND	0.023 ± 0.00
37	n-Decanoic acid	72.6	0.065 ± 0.02 ^b^	0.063 ± 0.02 ^ab^	ND	0.257 ± 0.14 ^a^	ND	0.054 ± 0.02 ^ab^	0.033 ± 0.03 ^ab^
38	Benzenepropanoic acid, α-(1-hydroxyethyl)	73.29	0.153 ± 0.06 ^a^	ND	0.286 ± 0.01 ^a^	ND	ND	ND	ND
39	9-Decenoic acid	79.15	ND	ND	ND	0.0222 ± 0.01	ND	ND	ND
40	Benzoic acid	88.42	ND	ND	ND	ND	0.004 ± 0.00 ^a^	ND	0.154 ± 0.00 ^a^
**Terpenes**									
41	β-Phellandrene	12.64	ND	ND	ND	ND	0.002 ± 0.00	ND	ND
42	β-Pinene	15.93	ND	ND	ND	ND	0.012 ± 0.00 ^a^	ND	0.0048 ± 0.00 ^a^
43	β-Myrcene	16.02	ND	ND	ND	ND	ND	0.001 ± 0.00	ND
44	D-Limonene	17.56	0.012 ± 0.01 ^a^	0.003 ± 0.00 ^a^	ND	ND	0.299 ± 0.11 ^a^	0.026 ± 0.01 ^a^	0.096 ± 0.07 ^a^
45	γ-Terpinene	19.48	ND	ND	ND	ND	0.06 ± 0.01	ND	ND
46	o-Cymene	21.67	ND	ND	ND	ND	0.075 ± 0.01 ^a^	ND	0.007 ± 0.00 ^a^
47	trans-α-Bergamotene	36.17	ND	ND	ND	ND	ND	0.004 ± 0.00 ^a^	0.009 ± 0.00 ^a^
48	Terpinen-4-ol	37.81	ND	ND	ND	ND	0.008 ± 0.00 ^a^	0.005 ± 0.00 ^a^	ND
49	L-α-Terpineol	40.79	ND	ND	ND	ND	0.026 ± 0.00 ^a^	0.003 ± 0.00 ^a^	0.004a ± 0.00 ^a^
50	β-Bisabolene	41.29	ND	ND	ND	ND	0.145 ± 0.01 ^a^	0.023 ± 0.01 ^a^	0.042 ± 0.01 ^b^
**Aldehydes**									
51	Hexanal	12.62	ND	0.011 ± 0.00 ^a^	ND	ND	ND	ND	ND
52	Nonanal	29.6	ND	0.038 ± 0.01 ^a^	ND	0.014 ± 0.02 ^a^	ND	ND	ND
53	3-Furaldehyde	32	0.006 ± 0.00 ^b^	0.013 ± 0.01 ^ab^	ND	0.068 ± 0.063 ^ab^	ND	0.002 ± 0.00 ^ab^	0.017 ± 0.01 ^a^
54	2,4-Heptadienal, (E,E)	33.52	ND	0.025 ± 0.01	ND	ND	ND	ND	ND
55	Benzaldehyde *	34.11	0.046 ± 0.02 ^c^	0.172 ± 0.06 ^ab^	0.041 ± 0.00 ^bc^	0.17 ± 0.14 ^abc^	0.034 ± 0.00 ^abc^	0.007 ± 0.00 ^d^	0.013 ± 0.00 ^a^
56	2-Nonenal, (E)	35.11	ND	0.078 ± 0.03^a^	ND	ND	ND	ND	ND
57	2-Furancarboxaldehyde, 5-methyl-	37.11	0.007 ± 0.00 ^b^	0.023 ± 0.01 ^ab^	0.006 ± 0.00 ^ab^	0.049 ± 0.04 ^ab^	0.004 ± 0.00 ^b^	ND	0.013 ± 0.00 ^a^
58	2-Decenal, (E)	39.33	ND	0.107 ± 0.03	ND	ND	ND	ND	ND
59	Benzaldehyde, 4-methyl	39.58	ND	0.099 ± 0.04 ^a^	0.007 ± 0.00 ^a^	0.236 ± 0.18 ^a^	ND	0.014 ± 0.01 ^a^	0.027 ± 0.01 ^a^
60	2,4-Nonadienal, (E,E)	41.23	0.005 ± 0.00 ^a^	0.111 ± 0.03 ^a^	0.003 ± 0.00 ^a^	ND	ND	ND	ND
61	2-Undecenal	42.77	ND	0.057 ± 0.01	ND	ND	ND	ND	ND
62	3-Acetyl-1H-pyrroline	55.11	0.002 ± 0.00 ^a^	0.008 ± 0.00 ^b^	0.003 ± 0.00 ^a^	0.032 ± 0.02 ^a^	ND	ND	ND
**Benzenes**									
63	Toluene	9.97	ND	0.002 ± 0.00 ^a^	ND	0.008 ± 0.01 ^a^	0.002 ± 0.00 ^a^	ND	0.001 ± 0.00 ^a^
64	Pyridine	19.4	ND	ND	ND	ND	ND	ND	ND
65	Ether, 3-methyl-2-butenyl o-tolyl	16.07	ND	ND	ND	0.0383 ± 0.03	ND	ND	ND
66	Benzene, 1,1’-(1,2-cyclobutanediyl)bis-, cis	21.69	0.014 ± 0.00 ^a^	0.014 ± 0.01 ^a^	ND	ND	ND	ND	ND
67	Benzofuran, 2-methyl-	37.19	ND	ND	ND	ND	ND	0.007 ± 0.00	ND
68	Naphthalene	42.33	0.003 ± 0.00 ^a^	ND	0.013 ± 0.00 ^a^	0.006 ± 0.01 ^a^	ND	0.021 ± 0.01 ^a^	ND
69	Oxime-, methoxy-phenyl	43.03	0.028 ± 0.01 ^ab^	0.022 ± 0.01 ^a^	0.168 ± 0.00 ^a^	ND	0.019 ± 0.02 ^ab^	0.008 ± 0.01 ^ab^	0.025 ± 0.00 ^a^
70	Phenol, 2-methoxy	47.29	ND	0.019 ± 0.01^a^	ND	0.024 ± 0.01 ^a^	ND	ND	ND
71	Mequinol	47.57	ND	ND	ND	ND	ND	ND	0.002 ± 0.00
72	Benzene, 1,4-diethoxy-	51.56	ND	0.054 ± 0.02	ND	ND	ND	ND	ND
73	Creosol	53.49	0.069 ± 0.03 ^bcd^	0.796 ± 0.03 ^d^	0.022 ± 0.00 ^a^	0.033 ± 0.02 ^ab^	0.043 ± 0.01 ^abc^	0.002 ± 0.00 ^abc^	0.004 ± 0.00 ^abcd^
74	Phenol, 3-methyl	56.22	ND	ND	ND	ND	ND	0.005 ± 0.00	ND
75	Phenol, 4-ethyl-2-methoxy- *	57.54	0.222 ± 0.09 ^a^	0.59 ± 0.21 ^b^	0.283 ± 0.02 ^ab^	0.22 ± 0.13ab	0.033 ± 0.00 ^ab^	0.031 ± 0.01 ^ab^	0.215 ± 0.01 ^a^
76	p-Cresol	59.66	0.006 ± 0.00 ^b^	0.032 ± 0.02 ^ab^	0.028 ± 0.00 ^ab^	0.005 ± 0.00 ^ab^	0.001 ± 0.00 ^a^	0.003 ± 0.00 ^ab^	0.003 ± 0.00 ^ab^
77	Phenol, 3-ethyl	59.79	ND	0.013 ± 0.00 ^ab^	0.012 ± 0.00 ^a^	ND	ND	0.004 ± 0.00 ^ab^	ND
78	Phenol, 4-ethyl	64.74	0.293 ± 0.11 ^ab^	0.55 ± 0.197 ^abc^	0.21 ± 0.02 ^a^	0.741 ± 0.49 ^c^	0.017 ± 0.00 ^a^	0.013 ± 0.01 ^bc^	0.072 ± 0.00 ^c^
79	2-Methoxy-4-vinylphenol	66.73	0.08 ± 0.03 ^a^	0.1 ± 0.04 ^a^	1.428 ± 0.14 ^b^	0.446 ± 0.24 ^a^	ND	ND	0.004 ± 0.00 ^a^
**Others**									
80	2-Vinylfuran	11.7	ND	ND	ND	0.005 ± 0.00	ND	ND	ND
81	Methyl vinyl ketone	14.95	ND	ND	0.008 ± 0.00	ND	ND	ND	ND
82	Propene	15.6	ND	ND	ND	ND	ND	0.001 ± 0.00 ^a^	ND
83	Methanesulfonic anhydride	19.52	0.001 ± 0.00 ^a^	0.013 ± 0.00 ^a^	ND	0.002 ± 0.00 ^a^	ND	ND	ND
84	Furan, 2-pentyl	20.62	ND	ND	ND	0.035 ± 0.04 ^a^	ND	ND	ND
85	Cyclopentane, methyl	27.6	0.005 ± 0.00 ^a^	ND	ND	0.063 ± 0.06 ^a^	ND	ND	ND
86	Ethanone, 1-(2-furanyl)	33.89	ND	ND	ND	0.06 ± 0.07 ^a^	ND	0.005 ± 0.01 ^a^	ND
87	Cyclopropane, pentyl	35.27	0.007 ± 0.00 ^a^	0.019 ± 0.01 ^a^	ND	0.138 ± 0.13 ^a^	ND	ND	ND
88	Hexadecane	37.52	ND	0.007 ± 0.00	ND	ND	ND	ND	ND
89	Bicyclo[6.1.0]nonane, 9-(1-methylethylidene)	40.31	ND	0.019 ± 0.01	ND	ND	ND	ND	ND

The values represent the means ± standard deviation (*n* = 3) of relative abundance (ppm) referring to dodecanoic acid, methyl ester. Different superscripts in the same row indicate differences (*p* ≤ 0.05) among samples. * Differences (*p* ≤ 0.05) between artisanal and commercial *Tejuino* beverages. AR: El Nayar; AW: Wirikuta; AY: La Yesca; AZ: Zitacua; TC: Tepic Center; TL: León Street; TM: Music square; TT: Tecnólogico zone; RT: retention time (min); ND: not detected. E and EE indicate trans isomers with 1 or 2 doble bounds, respectively.

## Data Availability

The data presented in this study are available on request from the corresponding author.
